# A decision tree approach for investigating the background of research activity of community and hospital pharmacists in Mie Prefecture: a retrospective questionnaire-based survey

**DOI:** 10.1186/s40780-024-00385-3

**Published:** 2024-10-17

**Authors:** Yuki Asai, Yasushi Takai, Hideo Kato, Shun-ichi Hiramatsu, Yoshihiro Miki, Naoki Masuda, Takuya Iwamoto

**Affiliations:** 1 Department of Pharmacy, Faculty of Medicine, Mie University Hospital, Mie University, 2-174 Edobashi, Tsu, Mie 514-8507 Japan; 2 Department of Pharmacy, Mie Heart Center Hospital, 222-1 Ooyodo, Meiwa, Taki, Mie 515-0302 Japan; 3 Mie Pharmaceutical Association, 311 Shimazaki, Tsu, Mie 514-0002 Japan

**Keywords:** Research activity, Decision tree, Community pharmacist, Hospital pharmacist

## Abstract

**Background:**

The support system for research activities has not been sufficiently established in clinical settings. A survey should be conducted to identify the causes of low research activity among pharmacists and the characteristics of pharmacists who could serve as mentors to build a support system at the regional level.

**Methods:**

A retrospective cross-sectional survey was conducted with 156 pharmacists, including hospital and community pharmacists, who attended a webinar on research ethics held once a year in Mie Prefecture. Decision tree (DT) analysis was performed to extract the low research activities and pharmacists who could serve as mentors in research activities using independent factors identified by multivariate logistic regression analysis.

**Results:**

The questionnaire response rate was 72.4% (113/156), and most respondents were community pharmacists (81.4%). In the DT model, pharmacists who did not belong to academic societies (78%, 46/59) or those who belonged to one or two academic societies but had no certifications (100%, 5/5) had low research activities. Pharmacists who read papers more than once a month and had a nearby mentor (73%, 11/15) were more likely to become mentors in research activities.

**Conclusions:**

The combination of the number of academic societies and the presence of certifications determines the efforts in research activities. In addition to reading at least one paper monthly, the presence of a mentor for writing research papers may also be a crucial factor in becoming a mentor. The proposed DT model may be helpful in building a support system for research activities at the regional level.

## Background

The duty of clinical pharmacists is to address clinical questions through logical thinking processes; therefore, the capacity to perform research activities is required [1]. In Japan, the Yakugaku Kyoiku Model Core Curriculum was documented as “Inquiry into pharmaceutical issues and commitment to pharmaceutical research” and “Practice of research” [2]. A comprehensive Japanese survey revealed that 50.4% of pharmacists had presented at conferences, and 22.2% had accepted at least one research paper [3]. However, the support required for research activities, such as mentorship, financial support, and availability of academic papers, has been reported [3] indicating that the foundation for research activities has not been sufficiently established in clinical settings. Regional pharmacist residency programs in the United States provide trainees with opportunities to present their research results through posters and podium presentations [4]. The residency program promoted a two-fold increase in the ratio of research products to the number of residents [5], therefore, it may be necessary to establish a regional support system to stimulate research activities in Japan. To our knowledge, a foundation for research activities has not yet been established at the regional level for clinical practice in Japan.

In Mie Prefecture, there is only one university hospital and one pharmaceutical university, which is considerably fewer compared to urban areas. Furthermore, reports indicate that the number of pharmacists in Mie Prefecture is below the national average in Japan [6], suggesting that the educational infrastructure for clinical research may not be well-developed. These regional differences could potentially influence the research activities of pharmacists in Mie Prefecture compared to those in other parts of Japan. In response to this situation, the Mie Pharmaceutical Association established the “Research Activity Promotion Team” in February 2022 to support clinical research efforts among hospital and community pharmacists. This team held the “workshop on research design” and evaluated its effectiveness [7]. Decision tree (DT) analysis using a questionnaire from participants revealed that pharmacists who did not belong to any academic society without any certifications or accreditations related to pharmacy practices were associated with low research activities. However, this result may be attributed to several biases, including positive responses due to high motivation for research activities and a small sample size [7]. Furthermore, the factors influencing participants’ research activities may not represent the entire Mie Pharmaceutical Association. Therefore, a comprehensive survey to build a support system for research activities at the regional level is required to characterize pharmacists with low research effort and identify those who could serve as mentors.

In this study, we conducted a cross-sectional survey to investigate the background factors affecting research activities in Mie Prefecture. Based on the survey results, DT analysis was performed to characterize the pharmacists who required guidance on research activities and those who could potentially become mentors.

## Methods

### Study design

A retrospective cross-sectional survey was conducted with 156 pharmacists, including hospital and community pharmacists, who attended a webinar on research ethics held once a year. Community pharmacists affiliated with the Mie Pharmaceutical Association worked on this webinar for research activities. The webinar was held on December 6, 2023 with a 90-min lecture on research ethics and regulations. All the participants were members of the Mie Pharmaceutical Association.

### Questionnaire

The questionnaires were collected using Google Forms (Google, Mountain View, CA, USA). The URL for accessing the questionnaire was widely disseminated through e-mail to all attendees after the webinar. The survey results were tallied from December 6 to 28, 2023. To increase the response rate, a written reminder was sent to all attendees 1 week before the response deadline. An e-mail was sent to solicit responses by the deadline date. The questionnaire consisted of basic information, the presence of research activities since obtaining the pharmacist’s license, and the background influencing research activities. The details of these questions are listed in Table 1.

**Table 1:** Questionnaire contents

	Contents
Basic information	
1	Sex
	□ Male, □ Female
2	Age, years old
	□ 20 to 29, □ 30 to 39, □ 40 to 49, □ 50 to 59, □ 60 to 69, □ ≥ 70
3	Pharmacist experience, years
	□ < 1, □ 2 to 5, □ 6 to 10, □ 11 to 20, □ 21 to 30, □ ≥ 31
4	Workplace distribution
	□ Community pharmacy, □ General Hospital / Clinic, □ Others
5	Did you get pharmacy license after pharmacy school transitioned to a six-year curriculum?
	□ Yes, □ No
(Outcome)	
Presence of research activities since obtaining a pharmacist license	
1	Have you ever performed a presentation on the results of your research activities in conference?
	□ At least once a year, □ Once every few years, □ Once or less
2	Have you ever written a research paper as a first author on the results of your research activities?
	□ Yes, □ No
(Exploratory factor)	
Influencing background for research activities	
1	Are there any mentor for research activities in work place or neighborhood?
	□ Yes, □ No
2	Are there any mentor for writing a research paper in work place or neighborhood?
	□ Yes, □ No
3	Do you have any certifications related to pharmacy practice?
	□ Yes, □ No
4	How many academic societies (except for the Japan Pharmaceutical Association and the Japanese Society of Hospital Pharmacists) do you belong to?
	□ 0, □ 1, □ 2, □ 3, □ 4, □ ≥ 5
5	Are there any opportunities to read research papers (English or Japanese)?
	□ Multiple papers / month, □ About one paper / month, □ Not at all

### Outcome and definition

The outcomes were: 1) the opportunity to present the results of research activities at conferences and 2) the presence of a paper publication as the first author.

In a previous study [7], low research activities were defined as responders who answered “once or less” for the opportunity to present the results of research activities at a conference (Table 1).

There may be obstacles between presenting at conferences and writing research papers [8], therefore, conference presentations do not necessarily lead to paper publications. Consequently, pharmacists who could become mentors were identified as those who had published papers as first authors (Table 1).

### Statistical analysis

A total of 156 pharmacists attended the webinars. The required sample size for this population was calculated to be 112, assuming a 50% collection rate for each facility, a 95% confidence level, and a 5% margin of error.

The objective variables were set as follows: 1) low research activities and 2) pharmacists who could serve as mentors for research activities, whereas the explanatory variables included the influencing background for research activities (Table 1). Categorical variables were compared using the chi-squared or Fisher’s exact test based on an expected value of < 5 on a 2 × 2 contingency table. For the multivariate logistic regression analysis, the stepwise forward selection method was used to determine the final model, as the influencing factors for research activities remain incompletely elucidated. Multicollinearity was evaluated using the variance inflation factor for all factors included in the univariate analysis. Factors with a *p* value < 0.1 and a variance inflation factor < 5 were selected as explanatory variables for the multivariate logistic regression analysis. The cut-off value of age and pharmacist experience were determined by the respective median value. All statistical analyses were performed using SPSS Statistics version 27 (IBM Japan, Tokyo, Japan), and the significance level was set at *p* < 0.05.

### DT analysis

DT analysis was performed according to our previous study [9] based on the chi-squared automatic interaction detection algorithm. Here, the dependent variables were set as 1) low research activities or 2) pharmacists who could serve as mentors in research activities. Given that the chi-square automatic interaction detection algorithm cannot adjust for confounding factors, the independent variables used in the DT analysis were extracted from the risk factors identified in the multivariate logistic regression analysis. Thus, the explanatory variables were used as factors with *p* < 0.05 in multivariate logistic regression analysis. For sensitivity analysis, the DT model was re-constructed using the factors with *p* < 0.2 in the univariate analysis as explanatory variables. The accuracy rates were calculated. In the subgroup analysis, DT analysis was also performed, focusing on community pharmacists.

All DT analyses were performed using SPSS Decision Tree version 27 (IBM Japan, Tokyo, Japan).

## Results

### Respondents’ characteristics

The questionnaire response rate was 72.4% (113/156). As shown in Table 2, the participating pharmacists had a wide range of experience. Most respondents were community pharmacists (81.4%, 92/113) and had completed a fourth-year curriculum (77%, 87/113).

**Table 2:** Background characteristics of respondents

Factor		Respondents, n	Rate, %^a^
All (*n* = 156)		113	72.4
Sex	Male	62	54.9
	Female	51	45.1
Age, years old	20 to 29	7	6.2
	30 to 39	26	23.0
	40 to 49	28	24.8
	50 to 59	28	24.8
	60 to 69	17	15.0
	≥ 70	1	0.9
Workplace distribution	Community pharmacy	92	81.4
	General Hospital / Clinic	20	17.7
	Pharmaceutical wholesaler	1	0.1
Region in Mie prefecture	Yokkaichi	25	22.1
	Tsu	22	19.5
	Suzuka-Kameyama	18	15.9
	Matsuzaka	11	9.7
	Iga	11	9.7
	Ise	7	6.2
	Toba-Shima	4	3.5
	Kuwana	2	1.8
	Kihoku	1	0.9
	Kinan	1	0.9
	Others	11	9.7
Pharmacist experience, years	< 1	0	0.0
	2 to 5	8	7.1
	6 to 10	15	13.3
	11 to 20	32	28.3
	21 to 30	39	34.5
	≥ 31	19	16.8
Pharmacy school curriculum	Six-year	26	23.0
	Four-year	87	77.0

^a^(Response / all respondents) × 100

### Outcome

**Fig. 1:**
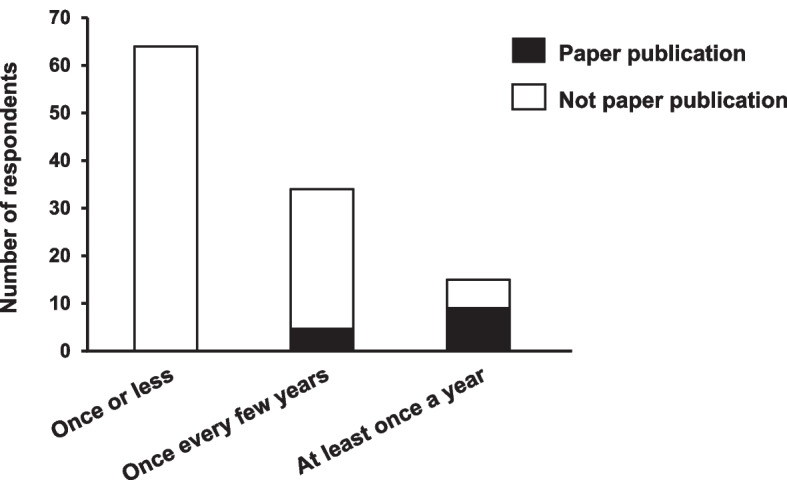
Relationship between frequent presentations in conferences and publications as respondents

Respondents’ research activities are shown in Fig. 1. For the experience of presentation at conferences, there were 64 respondents (57%, 64/113) in “once or less,” 34 respondents (30%, 34/113) in “once every few years,” and 15 respondents (13%, 15/113) in “at least once every year.” Of these respondents, 15% (5/34) in “once every few years” and 60% (9/15) in “at least once a year” had experience of paper publication.

### Influencing background for the low research activities

The influencing background for low research activities, defined as respondents who answered “once or less” for the opportunity to present the results of research activities at the conference, is shown in Table 3. Univariate analysis revealed that male sex (*p* = 0.007), workplace at community pharmacies (*p* = 0.001), absence of mentors for research activities (*p* = 0.008), absence of mentors for writing a research paper (*p* = 0.009), not obtaining certifications (*p* = 0.002), lack of membership in academic societies (*p* < 0.001), and opportunities to read papers less than once per month (*p* = 0.001) were associated with low research activities. In the multivariate logistic regression analysis, the adjusted odds ratios for workplaces at community pharmacies, did not obtain certifications, and did not have membership in academic societies were 4.72 [95% confidence interval [CI]: 1.105–20.122, *p* = 0.036], 8.96 (95%CI: 1.662–48.290, *p* = 0.011), and 4.46 (95%CI: 1.791–11.115, *p* = 0.001), respectively.

**Table 3:** Background related to low research activities

Factors	Univariate analysis				Multivariate logistic regression analysis		
	OR	95%CI	*p* value	VIF	Adjusted OR	95%CI	*p* value
Male	0.34	0.157–0.751	0.007^a^	1.174	-	-	-
Age (20 to 39 years old)	1.51	0.654–3.469	0.335^a^	3.873	-	-	-
Pharmacist experience (≤ 10 years)	2.00	0.751–5.329	0.161^a^	3.247	-	-	-
Workplace (community pharmacy)	5.72	1.922–17.031	0.001^a^	1.418	4.72	1.105–20.122	0.036
Graduated from a six-year pharmacy school	1.61	0.646–3.999	0.305^a^	5.863	-	-	-
Absence of mentors for research activities	2.88	1.299–6.385	0.008^a^	2.873	-	-	-
Absence of mentors for writing a research paper	2.91	1.285–6.590	0.009^a^	3.101	-	-	-
Not obtained any certifications	8.50	1.859–38.861	0.002^a^	1.141	8.96	1.662–48.290	0.011
Not a member of any academic societies	7.08	3.067–16.330	< 0.001^a^	1.606	4.46	1.791–11.115	0.001
Opportunities to read papers (< one / month)	5.72	1.922–17.031	0.001^a^	1.569	-	-	-

*OR* Odds ratio, *VIF* Variance inflation factor, *95%CI* 95% coefficient interval

^a^Chi-square test

### Influencing background for the pharmacists who can serve as mentors to research activities

The influencing background for pharmacists who can serve as mentors in research activities, defined as those who have published papers as the first author, is shown in Table 4. Univariate analysis revealed that workplaces at general hospitals/clinics (*p* < 0.001), the presence of mentors for research activities (*p* < 0.001), the presence of mentors for writing a research paper (*p* < 0.001), being a member of an academic society (*p* = 0.002), and opportunities to read papers more than once per month (*p* < 0.001) were involved in pharmacists who can serve as mentors for research activities.

**Table 4:** Factors influencing the potential for pharmacists to become future mentors in research activities

Factors	Univariate analysis				Multivariate logistic regression analysis		
	OR	95%CI	*p* value	VIF	Adjusted OR	95%CI	*p* value
Male	3.45	0.907–13.128	0.057^a^	1.170	-	-	-
Age (≥ 40 years old)	0.50	0.159–1.575	0.345^b^	3.881	-	-	-
Pharmacist experience (> 10 years)	0.40	0.120–1.337	0.156^b^	3.251	-	-	-
Workplace (General Hospital / Clinic)	9.67	2.858–32.693	< 0.001^b^	1.352	-	-	-
Graduated from a six-year pharmacy school	2.96	0.923–9.513	0.086^b^	5.716	-	-	-
Presence of mentors for research activities	15.2	3.199–72.368	< 0.001^b^	2.874	-	-	-
Presence of mentors for writing a research paper	18.8	3.917–89.758	< 0.001^b^	3.111	8.07	1.356–48.038	0.022
Obtained certifications	2.89	0.355–23.523	0.458^b^	1.144	-	-	-
Membership in academic societies	8.14	1.730–38.330	0.002^a^	1.589	-	-	-
Opportunities to read papers (≥ one /month)	60	11.57–311.27	< 0.001^b^	1.547	34.3	6.151–191.28	< 0.001

*OR* Odds ratio, *VIF* Variance inflation factor, *95%CI* 95% coefficient interval

^a^Chi-square test

^b^Fisher’s exact test

In multivariate logistic regression analysis, the adjusted odds ratios for the presence of mentors for writing a research paper and opportunities to read papers more than once /month were 8.07 (95%CI: 1.356–48.038, *p* = 0.022) and 34.3 (95%CI: 6.151–191.28, *p* < 0.001), respectively.

### DT analysis

**Fig. 2:**
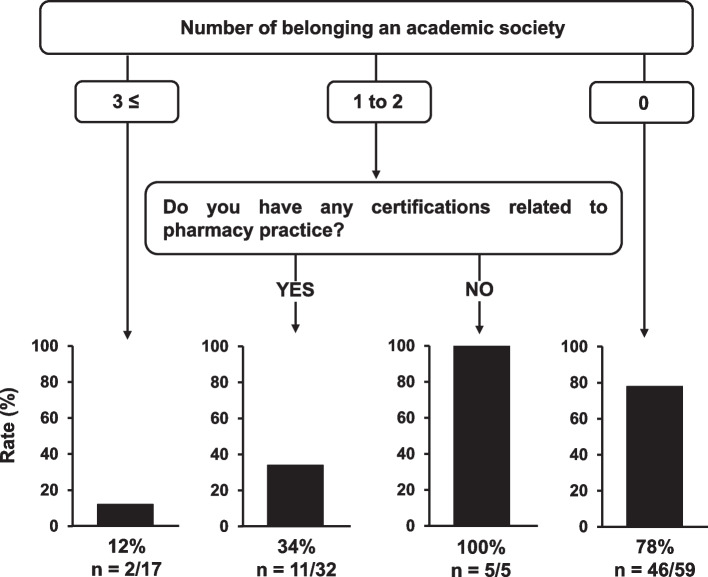
Decision tree model for predicting the pharmacists with low research activity. The low research activities were defined as responders who answered “once or less” for the opportunity of presentation on the results of research activities in conference

In the DT analysis, pharmacists who did not belong to academic societies (78%, 46/59) or members of one or two societies who did not have certification (100%, 5/5) were seldom involved in research activities. In contrast, pharmacists belonging to more than three academic societies were highly involved in clinical research (12%, 2/17) (Fig. 2). The accuracy of the DT model is 77.0%. The sensitivity analysis also confirmed that the same DT models were constructed.

**Fig. 3:**
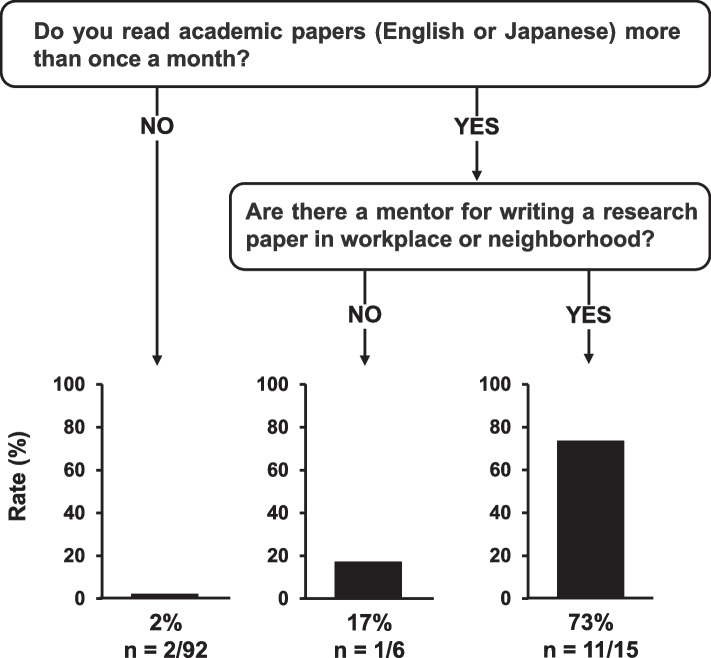
Decision tree model for predicting the pharmacists who become future mentors for research activities. The pharmacists who could become future mentors were identified as those who had published papers as first authors

As shown in Fig. 3, pharmacists who read papers more than once per month and had a mentor nearby were likely to publish papers as the first author and serve as mentors for research activities (73%, 11/15). The accuracy of the DT model is 93.8%.

**Fig. 4:**
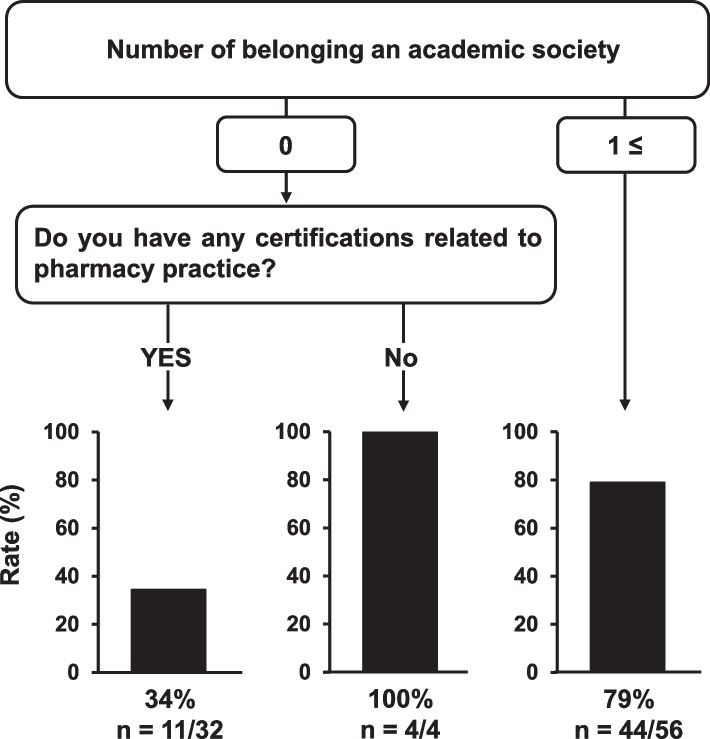
Decision tree model for predicting community pharmacists with low research activity. This decision tree model focused on community pharmacists (*n* = 92) as part of a subgroup analysis. Low research activity was defined as responders who answered “once or less” regarding their opportunity to present the results of research activities at conferences

In the subgroup analysis (Fig. 4), community pharmacists who were not affiliated with any academic societies and had not obtained any certifications (100%, 4/4) or those affiliated with one or more academic societies (79%, 44/56), exhibiting a high proportion of low effort. In contrast, pharmacists who were not affiliated with academic societies but held qualifications showed a lower proportion of low effort, at 34% (11/32). The accuracy of the DT model was 75.0%. A DT model for pharmacists who could serve as mentors in research activities could not be constructed, as there were only six pharmacists who had published papers as first authors.

## Discussion

The DT model showed that the combination of the number of academic societies to which they belong and the presence of certifications determined the number of research activities, defined as the frequency of presentations at conferences. In addition to reading at least one paper monthly, the presence of a mentor for writing research papers may be a crucial factor for writing papers and becoming a future mentor for research activities. Although there is no consensus regarding the accuracy of the prediction model, the accuracy of the DT models was comparable to those reported in previous studies [9–11]. Moreover, the branched characteristics of the two DT models were identical to those of the sensitivity analyses, suggesting that the DT model was well-validated.

The number of responses received for this study was 113, which was higher than the calculated required sample size. In the present questionnaire, 81.4% of the respondents were community pharmacists (Table 2). Although community pharmacists affiliated with the Mie Pharmaceutical Association have a duty to attend the webinar on research ethics held once a year, hospital pharmacists have several opportunities in their respective hospitals, such as e-learning systems and lectures; therefore, hospital pharmacists may have less need to take this webinar. It was speculated that there was substantial polarization in workplace distribution among the respondents. Although several studies on research activities have mostly included hospital pharmacists as respondents [3, 12, 13], few reports have been specific to community pharmacies. Recently, the number of community pharmacists has increased, and regional maldistribution has improved [14], suggesting that the pharmacists’ characteristics for research activities identified in the present study would be helpful in building a support system at the regional level.

In the DT model for predicting low research activities (Fig. 2), the number of academic societies was likely associated with the presentation rate. As membership and registration for conferences are required for presentations in academic societies, it may be reasonable for the number of members of an academic society to be the first node in the DT model. Although this branch was also observed in our previous report [7], it was possible to categorize the number of academic societies in detail by increasing the sample size. Multivariate logistic regression analysis revealed that working in a community pharmacy was an independent influencing factor for low research activities, defined as one or fewer opportunities for presentations at conferences (Table 3). Sagawa et al. [15] reported barriers to research activities, such as the lack of mentors for clinical research and limited opportunities for discussions in community pharmacies. Moreover, the experience of conference presentations among hospital pharmacists was 2.6 times higher than that of community pharmacists [3], suggesting that pharmacists in community pharmacies require support for their research activities. Therefore, DT analysis focused on community pharmacists was conducted (Fig. 4), demonstrating that the absence of any certifications related to pharmacy practice had a greater impact on low effort than the number of society affiliations. In general, because obtaining certifications related to pharmacy practice often requires presentations at academic conferences, this result seems reasonable. Considering these results, it is likely that a support system for obtaining certifications related to pharmacy practice will be necessary to promote research activities.

The publication rate of studies presented at five national pharmacy-associated meetings in the United States was only 19.8% [8] therefore, there may be obstacles between presenting at conferences and writing research papers. It can be inferred that it may be difficult to publish a paper without a mentor, as supported by the multivariate logistic regression analysis (Table 4). Moreover, it might be reasonable to extract the frequency of reading academic papers as an independent factor because of the need to collect reference articles while writing a paper. Reading academic papers allows researchers to expand their knowledge by engaging with the work of others, thereby acquiring the background information and new perspectives necessary for their own research. This process facilitates the generation of new research ideas, increasing the likelihood of writing papers. However, the correlation between reading and writing papers varies among individuals, and this study has not clarified this relationship. Therefore, future research should investigate the content of papers read regularly to enable a more detailed analysis. According to the DT model for predicting the experience of paper publication as the first author, it was speculated that pharmacists who read at least one paper per month and had a mentor for writing papers close to them were more likely to have publications and could be mentors. It has been reported that mentor training in research activities is beneficial in promoting research activities [16]. Therefore, a mentor training program should be established by a Research Activity Promotion Team for pharmacists using the characteristics identified in this DT model.

The present study had some limitations. First, since this survey was limited to the Mie Prefecture, it may not reflect the current situation in other areas. Although additional surveys in other areas should be considered, there may be no major discrepancies because of the reproducibility of background factors in the experience of writing papers in a comprehensive survey in Japan [3]. Second, since the survey was anonymous, measures were taken to ensure that respondents could not be identified, and detailed information on participants’ institutions was not collected. Consequently, we could not evaluate whether the findings of this survey are dependent on any particular institution. Third, because the pharmacists who attended this webinar might have a high awareness of research activities, pharmacists with little interest in research activities might not be included in the analysis. Fourth, because the questionnaire was a self-reported survey, bias might have existed in its accuracy. Fifth, factors influencing research activities may differ by population. Sixth, the frequency and number of published papers may be important conditions for mentor recruitment, but this has not yet been investigated. Seventh, 14 respondents indicated that they have published a paper that is problematic in terms of robustness to conduct a multivariate logistic regression analysis. Finally, because most respondents graduated from 4-year pharmacy school curriculum, there is a possibility of bias due to the educational system, such as the Yakugaku Kyoiku Model Core Curriculum. Despite these limitations, the strength of this study was that it was limited to Mie Prefecture, which leads to the promotion of research activities by a bottom-up strategy at the regional level.

## Conclusions

This study revealed that future workshops and/or lectures should target pharmacists who are not members of academic societies or have not obtained certifications related to pharmacy practice in order to promote research activities. Furthermore, pharmacists at hospitals and community pharmacies where there is a mentor for writing papers and habitually reading academic papers could potentially serve as future mentors for research activities through training. We believe that the present DT model may be helpful in building a support system for research activities in Mie Prefecture.

## Data Availability

The data that support the findings of this study are available from the corresponding author upon reasonable request.

## Abbreviations

CI: Confidence interval
DT: Decision tree
